# Direct high-resolution X-ray imaging exploiting pseudorandomness

**DOI:** 10.1038/s41377-023-01124-3

**Published:** 2023-04-06

**Authors:** KyeoReh Lee, Jun Lim, Su Yong Lee, YongKeun Park

**Affiliations:** 1 Department of Physics, Korea Advanced Institute of Science and Technology, Daejeon, 34141 Republic of Korea; 2 KAIST Institute for Health Science and Technology, Korea Advanced Institute of Science and Technology, Daejeon, 34141 Republic of Korea; 3 Pohang Accelerator Laboratory, Pohang University of Science and Technology, Pohang, Kyungbuk 37637 Republic of Korea; 4 Tomocube Inc, Daejeon, 34051 Republic of Korea

**Keywords:** Imaging and sensing, X-rays

## Abstract

Owing to its unique penetrating power and high-resolution capability, X-ray imaging has been an irreplaceable tool since its discovery. Despite the significance, the resolution of X-ray imaging has largely been limited by the technical difficulties on X-ray lens making. Various lensless imaging methods have been proposed, but are yet relying on multiple measurements or additional constraints on measurements or samples. Here we present coherent speckle-correlation imaging (CSI) using a designed X-ray diffuser. CSI has no prerequisites for samples or measurements. Instead, from a single shot measurement, the complex sample field is retrieved based on the pseudorandomness of the speckle intensity pattern, ensured through a diffuser. We achieve a spatial resolution of 13.9 nm at 5.46 keV, beating the feature size of the diffuser used (300 nm). The high-resolution imaging capability is theoretically explained based on fundamental and practical limits. We expect the CSI to be a versatile tool for navigating the unexplored world of nanometer.

## Abstract (web summary)

High-resolution X-ray sample field image is directly retrieved in a single shot using the mathematical relations of spatially pseudorandom (i.e., speckle) fields.

## Graphical abstract

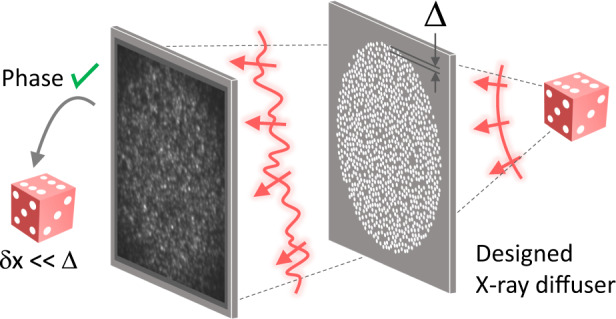

## Introduction

Making an X-ray imaging lens has been a challenging quest. Because of the near-unity refractive indexes of materials in X-ray, collecting scattered rays through refractive optics is difficult^1^. Although Fresnel zone plates have been used, their low coupling efficiency and technical difficulties in high-resolution zone plate fabrication have been problematic, particularly for hard X-rays^2,3^.

Lensless X-ray imaging methods have therefore been investigated^4^. The main concept of these methods is applying a numerical, instead of physical, lens by measuring the sample diffraction field. However, the complex-valued sample field is not a measurable quantity. Although an image sensor can measure the intensity of a diffracted sample field, its phase information is lost during acquisition; reconstruction of the lost phase has been a major challenge in lens-free X-ray imaging^5^.

Additional constraints should be introduced a priori to determine the correct phase values^6,7^. A successful example is coherent diffractive imaging (CDI), which utilizes the sample support as a constraint^8,9^. With CDI, the sample field is reconstructed from a far-field diffraction pattern using iterative algorithms^10,11^. High-resolution phase imaging and a simple setup are the advantages of CDI in materials^12,13^ and biological studies^14–18^. Nevertheless, sample support constraints often limit the utility and feasibility of CDI. Because small support deviations may induce severe artifacts, defining proper support is critical in CDI^19,20^. Such strict support criteria hinder the application of CDI for more general samples with ambiguous boundaries or extended structures. Although the support criteria can be effectively mitigated by inserting an additional modulator after the sample^21,22^ or introducing a known probe instead of sample support, the convergence and reliability of the results still depend on sample properties such as support looseness, resolution, size, or phase variation.

Ptychography is another lensless imaging technique; it utilizes multiple acquisitions instead of a sample support constraint. In ptychography, a sample is laterally scanned using an X-ray probe, and position-dependent far-field diffraction patterns are obtained^23,24^. With sufficient overlap between adjacent probe beams, the correct phase solution can be determined without additional constraints^25^. Owing to the sample generality, ptychography is a promising solution for extended or confluent samples. Various samples have been investigated for two-^26–29^ and three-dimensions^30,31^. However, the requirement of multiple acquisitions results in limitations in applicability; dynamic or degradable samples may not be applicable. Sample radiation damage can be severe, particularly for biological samples and X-ray free-electron laser (XFEL) applications^32,33^. Although reducing the X-ray dose and portions of overlap may prevent radiation damage, it may limit the signal-to-noise ratio and image resolution^34,35^.

**Fig. 1:**
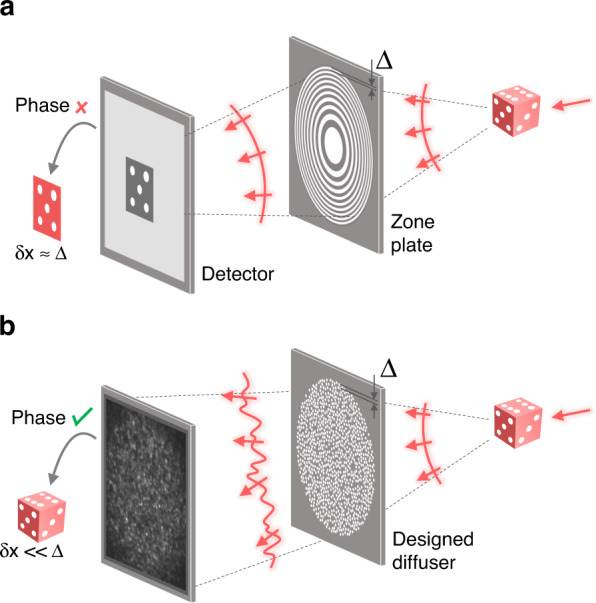
Designed diffuser as X-ray imaging lens. **a** Schematic of full-field transmission X-ray microscopy. The attenuation (amplitude) map of a sample is measured. The system resolution is defined by the outermost zone width of the zone plate (δ*x* ≈ Δ). **b** Schematic of the proposed CSI method. The zone plate is replaced with a designed diffuser. The complex-valued sample field is measured. The system resolution is finer than the hole size of the diffuser (δ*x* « Δ)

Thus, we present coherent speckle-correlation imaging (CSI) in X-ray. In CSI, we transform the sample field into a random speckle before the measurement using an X-ray diffuser^36^. Although the phase of the speckle is immeasurable either, the complex-valued sample field can be uniquely reconstructed from the magnitude image through the pseudorandomness of speckle^37–40^. Our CSI exploits a designed X-ray diffuser after the sample instead of a zone plate (Fig. 1), achieving a 13.9-nm image resolution at 5.46 keV; this is far below the feature size of the diffuser used (300 nm). The oversampling ratio and setup requirements are discussed in relation to the image resolution and sample field of view (FOV) acquired.

## Results

### Experimental setup

**Fig. 2:**
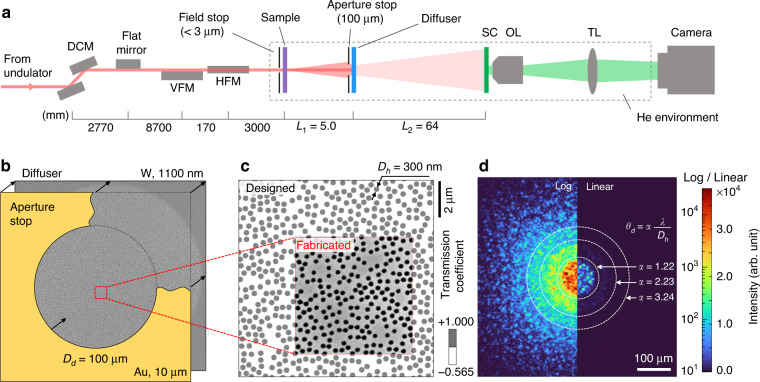
Experimental setup and diffuser. **a** Schematic of the experimental setup. DCM double-crystal monochromator, VFM/HFM vertical/horizontal focusing mirror, SC scintillator, OL/TL objective/tube lens (see Methods). **b** Scanning electron microscopy (SEM) image of the diffuser. The diameter of the diffuser (*D*_*d*_ = 100 μm) defined by the aperture stop placed immediately in front of the diffuser. **c** Magnified SEM image of the diffuser and designed transmission function. A 300-nm (*D*_*h*_) hole is randomly placed on the 1100-nm sputtered tungsten layer. **d** A measured speckle pattern without a sample is shown at the logarithm (left) and linear (right) scales, various maximum deflection angle (*θ*_*d*_) criteria. α = 1.22 is used in this work (see Methods)

The experimental setup of the CSI is shown in Fig. 2a (see Methods). A coherent X-ray beam (5.46 keV) was used at the 9C beamline of the Pohang Light Source II (PLS-II). A field stop (<3.0 μm) is placed before a sample to maintain the spatial coherence and eliminate ambient signals outside the sampling domain. The diffuser is a sputtered tungsten layer on a silicon nitride membrane with 300-nm etched holes in unordered but known locations (Fig. 2b). The tungsten thickness and hole density are designed to minimize the unmodulated term (*q* = 0) of the diffuser and generate well-developed speckle patterns for a given photon energy (see Methods). The diameter of the diffuser is defined by the aperture stop (100 μm). Because the locations of the holes are predefined, the transmission function of the diffuser is known (Fig. 2c), and the output field for a given incident field can be calculated. An experimental speckle pattern without a sample is shown in Fig. 2d. We verified the hole size and tungsten thickness of the fabricated diffuser from the lobe sizes of the diffraction patterns, and the portion of the unmodulated term at the center, respectively. As intended, visible speckle grains without a strong unmodulated term are shown even in the linearly scaled image.

Nonetheless, potential errors in diffuser fabrication can cause discrepancies between the calculated and measured speckle patterns, which may degrade the field retrieval fidelity of CSI. For instance, the diffuser hole size can be varied due to slight undercut (or overcut) in fabrication process. Fortunately, we find the granular patterns of speckles do not significantly change from the hole size variation (Supplementary Fig. S1). This is because the speckle pattern on the detector is composed of the far-field diffractions of holes, and the phase profile of each hole diffraction is primarily related to the hole position the rather than the hole size.

Potential errors in diffuser alignment can also degrades the field retrieval fidelity of CSI. Diffuser misalignment refers to the discrepancy between the expected and actual diffuser hole positions, which causes significant decorrelation in the speckle patterns. In order to minimize the error, we introduced a numerical finetuning process to reflect the actual position and roll angle of the diffuser. Because even a slight deviation significantly degrades the field retrieval fidelity (Supplementary Fig. S2), the correct position and roll angle could be determined precisely by maximizing the field retrieval fidelity. We also compensated the blurring effect of the scintillator through point spread function (PSF) deconvolution. The PSF of the detection system is measured by providing a sub-diffraction-limit X-ray focus on the scintillator (see Methods). Note that this calibration process is required once after the optical system setup.

### Reconstruction flow

The CSI field reconstruction flow consists of two major steps: the initial guess of a sample field from the speckle-correlation scattering matrix (SSM); and the error reduction algorithm that retrieves the final solution using the initial guess^36^. In this work, we employ the amplitude flow (AF) for the error reduction step^41^. Please find the detailed description of both steps in Methods.

As summarized in Supplementary Fig. S3, the full-reconstruction flow of CSI only requires the measured intensity speckle, predefined transmission matrix (TM), and the pseudorandomness of speckle. The TM is calculated through the transmission function of the designed diffuser (Fig. 2c), and the free-propagation distances before (*L*_1_) and after (*L*_2_) the diffuser (Fig. 2d). Random X-ray diffusers such as sandpapers^42,43^ therefore cannot be utilized for CSI unless their transmission function is pre-calibrated. Note that the sample field cannot be retrieved from the simple TM inversion because we cannot measure the phase of speckle field as well. Therefore, it should be emphasized that the TM-inversion-based imaging methods utilizing spatially incoherent illuminations^44,45^ or additional phase-measuring techniques^46,47^ are fundamentally different from CSI. Instead, we exploit the pseudorandomness of speckle in CSI. It is the core idea that grants the uniqueness of solution, and the global convergence of field reconstruction algorithm. It allows a speckle field to be considered as Gaussian random variables, which is the common prerequisite of the Isserlis’ theorem in the SSM construction (see Methods) and the stability of AF iteration (see Methods)^41^. The entire reconstruction took 40–70 s using a GPU (GeForce GTX 1080 Ti, NVIDIA Corp.).

### Field retrieval results

**Fig. 3:**
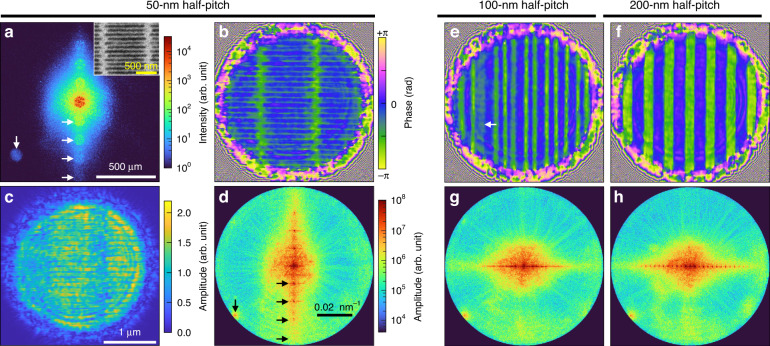
Retrieved sample fields and analysis of resolution. **a**–**d**, Retrieved sample field of 50-nm half-pitch grating. The measured speckle pattern (**a**), retrieved phase (**b**), amplitude image (**c**), and reciprocal space (**d**) are shown. An SEM image of the grating is shown in the subset of **a**. Horizontal arrows indicate a sample diffraction on the speckle pattern (**a**) and the corresponding sample signal (**d**). Vertical arrows indicate a consistent noise on the speckle pattern (**a**) and the corresponding noise signal (**d**). **e**–**h** Retrieved sample field of 100- and 200-nm half-pitch gratings. Phase images (**e** and **f**) and reciprocal spaces (**g** and **h**) are shown. Leftward arrow of **e** indicates the collapsed line of the 100-nm half-pitch grating sample

Grating structures patterned on a sputtered tungsten layer (700-nm thickness) were imaged for the first CSI demonstration using X-rays. A measured speckle pattern for a 50-nm half-pitch grating is shown in Fig. 3a. We accumulated one-hundred camera frames to enhance the signal-to-noise ratio (SNR) without saturation. A single frame took 250 ms; thus, the total acquisition time was 25 s. A total of ~1.6 × 10^11^ photons were used, or in other words, ~1.6 × 10^9^ photons per frame.

The corresponding sample fields retrieved are shown in Fig. 3b, c. The phase image clearly visualizes the grating structures, including the uneven etched depth of the lines (Fig. 3b). The amplitude image provides less contrast than the phase image^4^ (Fig. 3c). Diffraction from the field stop is observed in both the amplitude and phase images. The reciprocal space of the sample field is shown in Fig. 3d. Higher diffraction orders of the grating are easily observed, implying the high-resolution-imaging capability of CSI. The results for the 100- and 200-nm half-pitch gratings are also shown in Figs. 3e–h. Slight aperiodicity and some collapsed lines (arrow in the figure) of the 100-nm half-pitch grating are observed (Fig. 3e). Well-established lines are observed for the 200-nm half-pitch grating (Fig. 3f). The tungsten parts consistently show a delayed phase of −2 rad, which agrees with the theoretical value for a 700-nm-thick tungsten layer^48^. Note that the measured phases are negative values here because the refractive index of tungsten is smaller than unity.

The sampling domains in the real and reciprocal spaces are set based on the diameters of the stops used. The real space domain is set to a 3-μm diameter (*D*_*s*_) slightly larger than the field stop. The reciprocal space is bandlimited by the numerical aperture (NA) of a given system 1/2*D*_*d*_/*L*_1_ = 0.01, where *D*_*d*_ is the diffuser diameter (100 μm). Although CSI does not utilize any support constraint in the field retrieval algorithm, the two stops are still introduced in the setup to control the number of incident optical modes, and to minimize the unwanted signals. Note that conventional imaging systems (e.g., professional cameras) often have apertures for the same roles.

The sampling domains, however, can be set regardless of the experimental configuration. If we set the sampling domain to be smaller than the actual sample signal, the signal outside the defined domain cannot be comprehended and is regarded as noise during the reconstruction process (Supplementary Fig. S4). Similarly, if we set a larger sampling domain, the empty field will be retrieved as excess (Supplementary Fig. S5). Such mismatched settings are inefficient in terms of sampling and calculation speed but can be useful depending on the application. For example, low-NA reconstruction would be preferred for the optic alignments because of its faster reconstruction speed. This unconstrained sampling is a major advantage of CSI compared to the CDI-based imaging methods^8,21^ (Supplementary Fig. S6).

The CSI reconstructs the optical field regardless of the sample. Therefore, a defocused sample field is naturally observed if there were an offset in the sample axial position. The sample fields shown in Fig. 3 are, however, refocused onto the sample surface through numerical propagation (28-μm upstream) for clearer visualization. All the intermediate results are provided in Supplementary Figs. 7–9.

### Acquired image resolution

To quantify the spatial resolution, we calculated the Fourier ring correlation (FRC) of the retrieved sample fields. We randomly divided 100 frames of the measured speckle images into two sets of 50 frames, and accumulated them separately. Two individual sample fields were retrieved, and the FRC was calculated between them^49,50^. Ten FRC results were calculated for the different permutations and averaged. A consistent noise signal is observed in the lower-left corner of the speckle pattern, creating a high-frequency signal in the reciprocal space (Fig. 3a, d, the vertical arrows). We reject the term in the FRC calculation to prevent false high correlations (Supplementary Fig. S10).

**Fig. 4:**
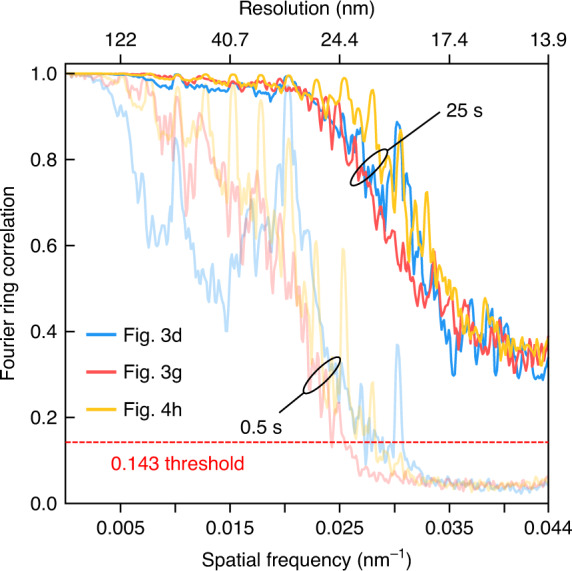
Fourier ring correlation (FRC) results. The FRC results of the retrieved grating results. FRCs of both long (25 s, bold color) and short (0.5 s, light color) acquisition times are compared

The FRC results are shown in Fig. 4. The FRCs of the three grating samples similarly decay as the spatial frequency increases. Based on the 0.143 threshold^51^, all three grating samples fully exploit the NA of the imaging system with a corresponding resolution $\delta x = 0.61\lambda /{{{\mathrm{NA}}}}$ of 13.9 nm, which corresponds with the reciprocal spaces showing the scattering signal extension to the aperture edge (Fig. 3d, g, h). We can identify the high-angle sample diffraction signal in the speckle image (Fig. 3a, the horizontal arrows), which is directly related to the high-frequency signal in reciprocal space (Fig. 3d, the horizontal arrows).

However, the sample-field resolution depends on the SNR. Weak sample scattering signals or large detection noise can reduce the effective spatial bandwidth of a retrieved sample field. To demonstrate this, we randomly selected one frame per set (instead of 50) and calculated the averaged FRC using the same sequence. This calculation represents a shorter acquisition time (0.5 s), exhibiting a lower SNR. As expected, the corresponding FRC results decay faster and cross the resolution threshold at ~0.025 nm^−1^, indicating a resolution of 24.4 nm (Fig. 4, light color).

### Resolution limit

According to the experimental results in Fig. 3a–h, the retrieved sample resolution (13.9 nm) is far below the diffuser hole diameter (*D*_*h*_ = 300 nm). This is a significant advantage that mitigates the practical difficulties in the fabrication process, especially compared to the X-ray microscopy using a zone plate.

Then, a question arises: what is the best resolution that can be achieved by a given diffuser? We found the resolution limit of the present method is derived from the oversampling criterion of the reconstruction algorithms,

1 $${\updelta}x_{\min } = \frac{1}{2}D_h\frac{{D_s}}{{D_d}}\left( {\sqrt {\gamma _{\min }} - 1} \right)$$

where *D*_*s*_ is the diameter of the sample FOV, and *γ*_min_ is the minimum oversampling ratio for stable field retrieval (see Methods). Equation (1) is proportional to *D*_*h*_. A smaller *D*_*s*_ has a finer resolution limit, which makes sense in terms of the space-bandwidth product (SBP) conservation. At *γ*_min_ = 4, an empirically known minimum value for noiseless simulations^36,39,41^, the ideal resolution limit of a given diffuser becomes ${\textstyle{1 \over 2}}D_hD_s/D_d$.

From Eq. (1), the resolution limit of the current system is 7.4 nm (*D*_*h*_ = 300 nm, *D*_*d*_ = 100 μm, *D*_*s*_ = 3 μm, and *γ*_min_ = 7), smaller than the experimentally demonstrated resolution (13.9 nm). This is derived from two additional practical criteria related to the resolution and size of the detection system (see Methods). We need to carefully choose *L*_1_ and *L*_2_ for a grain size and speckle pattern acceptable for the detector.

**Fig. 5:**
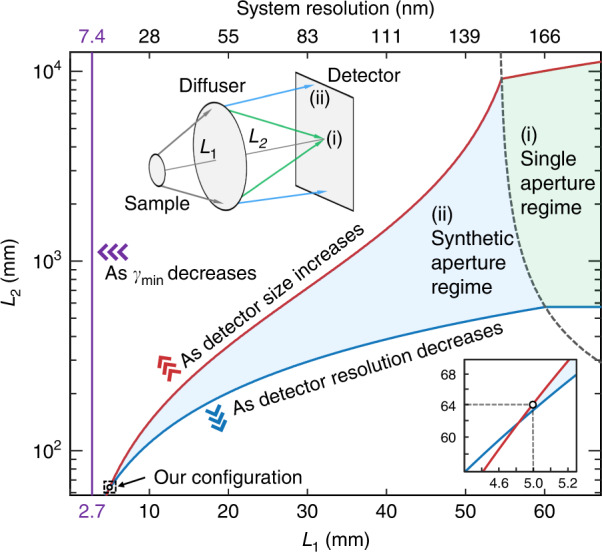
Resolution criteria on the *L*_*1*_*-L*_*2*_ plane. Fundamental (purple line) and two practical (red and blue lines) criteria for *L*_1_ and *L*_2_ for *γ*_min_ = 7 we set. The *L*_1_ and *L*_2_ pairs in the shaded area are available. The three-bracket arrows indicate the dependency of the boundary lines on the parameters. The experimental configuration is denoted as a circle (subset below). The synthetic aperture regime should be considered for the high-resolution imaging (blue shaded area). The black dashed line is the boundary between the two regimes, defined by Eq. (14). The inset figure visualizes the difference between the two regimes. See Methods for details

One fundamental and two practical criteria are plotted on the *L*_1_-*L*_2_ plane in Fig. 5. The shaded area indicates the available *L*_1_ and *L*_2_ satisfying all criteria. The detector resolution and size criteria provide the lower and upper bounds of *L*_2_, respectively. The minimum available *L*_1_ (resolution) is defined by these two practical criteria rather than the fundamental criteria. We may approach the resolution limit by increasing the detector size or decreasing the detector resolution (Fig. 5). In this work, the experimental parameters such as *L*_1_, *L*_2_, field stop diameter (<*D*_*s*_), and aperture stop diameter (=*D*_*d*_) were chosen based on this theoretical analysis.

### Imaging large FOV through stitching

To demonstrate the versatility of CSI, we measured samples with an extended FOV by stitching multiple holographic images. The samples were scanned by moving the stage along the hexagonal lattice in 1.8-μm steps. To horizontally cover the ~21-μm sample field, 91 scanning points were used. For fast scanning, we used a shorter acquisition time (250 ms) per scanning point, but with a reduced SNR and resolution (Fig. 4). The total scanning sequence takes approximately 35 s, excluding the data transfer time.

**Fig. 6:**
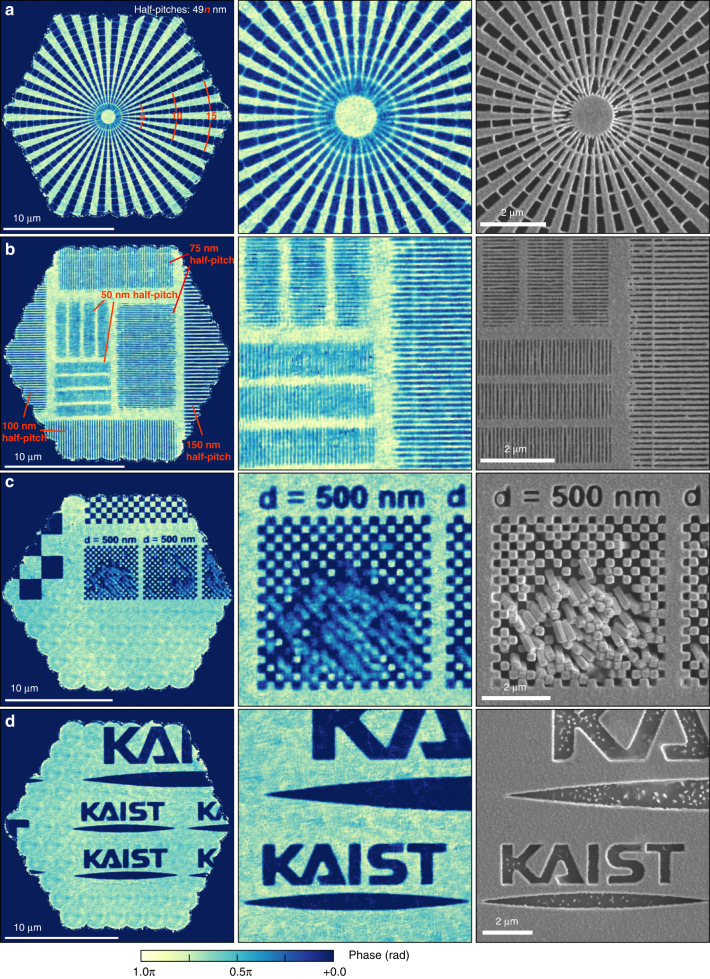
Stitched phase images. **a**–**d** Full-field (left) and magnified (right) images are shown for various samples. 91 hexagonal scanning points are used with a 1.8-μm step size. Identical sample is observed via scanning electron microscope (SEM) to provide references

A siemens star, grating arrays, checkerboard patterns, and university logos patterned on the same sputtered tungsten layer (700-nm thickness) were imaged (Fig. 6). The phase distributions of aperiodic and complex shapes were reconstructed well, as shown by the collapsed checkerboard (Fig. 6c) and letters (Fig. 6c, d). Fine structures were consistently reconstructed (Fig. 6c, d). Nevertheless, A slight degradation in quality can be found owing to the shorter acquisition time (e.g. 50-nm half-pitch grating in Fig. 6b).

Unlike in ptychography, the sample fields are retrieved individually before the stitching process in CSI. This may be advantageous if there are practical errors in sample scanning positions. We experienced significant position errors in sample scanning stages, and that is the reason of the irregular-shaped images in Fig. 6 (see Supplementary Fig. S11). The actual scanning positions are numerically adjusted based on the image correlation between adjacent scanning points.

Nonetheless, compared to ptychography, the insertion of a diffuser may not be favorable in some cases due to the inevitable photon loss from the diffuser. For instance, the calculated mean transmittance of used tungsten diffuser is 56.5% (see Methods). The reduced photon efficiency may become an issue because the spatial resolution of X-ray imaging is frequently limited by the photon flux^34^. However, it may be alleviated by using an X-ray diffuser made of a material with a lower atomic number. In our particular setup, the photon efficiency can be significantly enhanced by replacing the scintillator-based detector with a photon-counting X-ray detector^52^.

## Discussion

We propose and experimentally demonstrate CSI in X-ray. A designed X-ray diffuser was used to transform a sample field into a speckle pattern. Based on the speckle pseudorandomness, SSM and AF algorithms were introduced to retrieve the high-resolution sample field from the measured speckle pattern. The CSI performance was demonstrated for various samples. The resolution limit and proper system configurations were theoretically explored for the given experimental parameters.

We expect the CSI to be readily applicable to various studies and extended to various samples, different modalities, and other X-ray energies. One promising extension is X-ray tomography^30,31^, which has the same principles as optical diffraction tomography^53^. Introducing a sample rotation mount enables field measurements in different sample orientations, providing a 3D refractive index distribution of the sample^54^. The single-shot feature of CSI may be advantageous for short or time-lapse measurements.

Another potential extension is the introduction of an XFEL source, which is essential for high-resolution X-ray imaging of biological samples avoiding radiation damage^34,35^. Owing to its single-shot and constraint-free features, CSI will be a superb imaging solution for XFEL, particularly for samples with ambiguous supports. An additional beam block may be required to prevent damage to the diffuser from X-ray pulses.

Without a sample, the CSI can be utilized to characterize the X-ray beam profile. The spatial profiles of both the amplitude and phase of X-ray beams can be achieved using the same setup and field reconstruction flow, which can be utilized to measure the optical transfer function or aberrations of X-ray optics^29,55^. Real-time characterization of the beam wavefront is also possible by reducing the retrieval image resolution. We expect CSI to be utilized as an alignment and maintenance tool for X-ray sources (e.g., alignment of focusing optics).

The mixed state of the incident field and its individual microstates can also be retrieved via multiple eigenvectors of the SSM (rather than one, see Method)^39^. Although similar to mixed-state ptychography^56,57^, the single-shot requirement of the speckle-correlation method may again be an advantage. The speckle-correlation-based imaging can be utilized even for spatially incoherent sources by exploiting the memory effect and aperiodicity of a speckle pattern^44^.

## Methods

### Experimental setup

The experiments were conducted at the 9C beamline of PLS-II in Korea. The schematic setup is shown in Fig. 2a. A double-crystal monochromator (DCM) and a flat mirror were utilized to select the proper X-ray energy (5.46 keV) and filter its harmonic frequencies, respectively. The X-ray energy was set to provide the π-phase delay for a portion of the diffuser (1100-nm thickness of tungsten). The Kirkpatrick–Baez (KB) mirror pair was used to increase the flux of the samples. The photon flux density is ~1.3 × 10^15^ photons/s/mm^2^ at the sample location. A field stop (<3.0 μm) was placed less than 0.1 mm upstream from the sample; this provides a photon flux of ~6.4 ×10^9^ per second for a field stop diameter of 2.5 μm. The spatial coherence is ensured by the slits before the DCM. Used horizontal and vertical sizes of the slit are 40 μm and 200 μm, and the corresponding focal spot size is 9.4 μm and 5.0 μm (full width at half maximum), respectively.

A sample was placed in linear stages (Q-545.140, Physik Instrumente GmbH) for sample scanning. A designed diffuser was placed *L*_1_ = 5.0 mm downstream from the sample. An aperture stop (100 μm) is installed less than 0.15 mm upstream from the diffuser. The field stop and aperture stop were created using a focused ion beam (FIB) on 10 μm thick gold films (AU-173174, The Nilaco Corp.). A Tb:LSO scintillator (Tb^3+^:Lu_2_SiO_5_, 11.2 μm thick, *λ*_sc_ = 542 nm) on a YSO substrate (Yb_2_SiO_5_, 170 μm thick) is used as an X-ray detector^58^. The scintillator was placed *L*_2_ = 64 mm downstream from the diffuser. An optical microscope composed of an objective lens (NA = 0.4, ×10, UPLSAPO10X, Olympus Corp.), a tube lens (*f* = 180 mm, TTL180-A, Thorlabs, Inc.), and an sCMOS camera (6.5 μm, 2048 × 2048, Zyla 4.2 PLUS, Oxford Instruments plc) was used for speckle pattern measurements. The corresponding pixel and camera sensor sizes on the scintillator are 650 nm and 1.33 × 1.33 mm^2^, respectively. The sample chamber was filled with helium to minimize X-ray scattering from air.

### X-ray diffuser design

In CSI, the diffuser should provide a speckle field that can be regarded as a Gaussian random variable. To achieve this, it is important to minimize the unmodulated term $\mathop {\iint}\nolimits_{S_d} {t_d\left( {x,y} \right)dxdy}$, where $t_d\left( {x,y} \right)$ is the transmission function of the diffuser, and *S*_*d*_ is the area of the diffuser. Because our diffuser is a binary modulator, it can be rewritten as $t_{{{\mathrm{W}}}}\left( {S_d - N_hS_h} \right) + N_hS_h$, where *N*_*h*_ is the number of holes, *S*_*h*_ is the area of a single hole, and *t*_W_ is the transmission coefficient of tungsten (Fig. 2c). Therefore, the unmodulated term can be minimized using

2 $$N_h = \frac{{t_{{{\mathrm{W}}}}}}{{t_{{{\mathrm{W}}}} - 1}}\frac{{S_d}}{{S_h}}$$

Note that *t*_W_ should be real and negative (or π-delayed) to satisfy Eq. (2). To confirm the *N*_*h*_, we measured the *t*_W_ by comparing the presence and absence of the tungsten layer, and found that *t*_W_ = −0.565. The magnitude of *t*_W_ is smaller than the expected value (0.625) calculated from the tungsten refractive index. This discrepancy may have originated from the additional scattering loss from the nanostructures of the sputtered tungsten^59^. Based on Eq. (2) and the calibrated *t*_W_, we designed a diffuser by randomly placing *N*_*h*_ holes in area *S*. The hold diameter (300 nm) is determined based on the fabrication tests from ZonePlates Ltd., UK., and the acceptable minimum distance between the hole centers (330 nm) were empirically determined from numerical simulations. We found that too long center-to-center distance leads to periodic (hexagonal) alignment of holes, which is inappropriate for the random diffuser. The mean transmittance of the diffuser can be calculated using ${\textstyle{1 \over {S_d}}}\mathop {\iint}\nolimits_{S_d} {\left| {t_d\left( {x,y} \right)} \right|^2dxdy}$, which is −*t*_W_ = 0.565 according to Eq. (2).

### PSF measurement

The PSF measurement was performed in the same beamline without focusing mirrors. A zone plate of 300-μm diameter and 60-nm outermost zone width was used with a 50-μm diameter central stop. The corresponding focal length of the zone plate is 79.2 mm, which is also the axial distance between the zone plate and scintillator. A 50-μm diameter order-sorting aperture (OSA) is placed between them. The lateral and axial positions of the zone plate and OSA were fine-tuned using precise linear stages (AG-LS25, Newport Corp.).

The size of the X-ray focal spot is 73.2 nm, which is much smaller than the diffraction limit of the optical microscope used (827 nm). We sufficiently attenuated the X-rays to avoid damage to the scintillator and saturation. The measured PSF and corresponding optical transfer function are shown in Supplementary Fig. S12.

### Speckle-correlation scattering matrix (SSM)

If a sample field ${\mathbf{x}} \in {\mathbb{\text{C}}}^N$ is converted into a speckle field ${\mathbf{y}} \in {\mathbb{\text{C}}}^M$ passing through the system, the transmission matrix (TM) ${\mathbf{T}} \in {\mathbb{\text{C}}}^{M \times N}$satisfies ${{{\mathbf{y}}}} = {{{\mathbf{Tx}}}}$, where *N* and *M* are the numbers of spatial modes of the sample and speckle fields, respectively. The SBP was calculated to quantify the number of modes^60^. For instance, *N* is the product of the areas of the real and reciprocal sample domains defined. For the results in Figs. 3 and 6, we have *N* = 43,003 and *M* = 778,414.

Because we cannot measure the phase of **y**, the measured speckle pattern becomes ${{{\mathbf{I}}}} = {{{\mathbf{y}}}}^ \ast \circ {{{\mathbf{y}}}}$, where $\circ$ denotes the element-wise product. Using the TM and the measured speckle pattern, SSM ${\mathbf{Z}} \in {\mathbb{\text{C}}}^{N \times N}$ can be calculated as

3 $$Z_{ij} = \frac{{\left\langle {{{{\mathbf{t}}}}_i^ \ast \circ {{{\mathbf{t}}}}_j \circ {{{\mathbf{y}}}}^ \ast \circ {{{\mathbf{y}}}}} \right\rangle - \left\langle {{{{\mathbf{t}}}}_i^ \ast \circ {{{\mathbf{t}}}}_j} \right\rangle \left\langle {{{{\mathbf{y}}}}^ \ast \circ {{{\mathbf{y}}}}} \right\rangle }}{{\left\langle {{{{\mathbf{t}}}}_i^ \ast \circ {{{\mathbf{t}}}}_i} \right\rangle \left\langle {{{{\mathbf{t}}}}_j^ \ast \circ {{{\mathbf{t}}}}_j} \right\rangle }}$$

where ${{{\mathbf{t}}}}_i$ is the *i*th column vector of the TM, and the bracket indicates the mean of the vector elements $\left\langle \cdot \right\rangle = {\textstyle{1 \over M}}\mathop {\sum}\nolimits_{i = 1}^M {}$. The column vector ${{{\mathbf{t}}}}_i$ is another speckle field ${{{\mathbf{t}}}}_i = {{{\mathbf{Te}}}}_i$, where ${{{\mathbf{e}}}}_i$ is the *i*th basis vector of the sample field ${{{\mathbf{x}}}} = \mathop {\sum}\nolimits_i^N {x_i{{{\mathbf{e}}}}_i}$.

Because all four vectors (${{{\mathbf{t}}}}_i^ \ast$, ${{{\mathbf{t}}}}_j$, ${{{\mathbf{y}}}}^ \ast$, and ${{{\mathbf{y}}}}$) in the first term of the numerator of Eq. (3) are speckle fields that can be considered Gaussian random variables^61^, we decompose the term using the Isserlis’ theorem:^62^

4 $$\left\langle {{{{\mathbf{t}}}}_i^ \ast \circ {{{\mathbf{t}}}}_j \circ {{{\mathbf{y}}}}^ \ast \circ {{{\mathbf{y}}}}} \right\rangle = \left\langle {{{{\mathbf{t}}}}_i^ \ast \circ {{{\mathbf{t}}}}_j} \right\rangle \left\langle {{{{\mathbf{y}}}}^ \ast \circ {{{\mathbf{y}}}}} \right\rangle + \left\langle {{{{\mathbf{t}}}}_i^ \ast \circ {{{\mathbf{y}}}}} \right\rangle \left\langle {{{{\mathbf{t}}}}_j \circ {{{\mathbf{y}}}}^ \ast } \right\rangle + \left\langle {{{{\mathbf{t}}}}_i^ \ast \circ {{{\mathbf{y}}}}^ \ast } \right\rangle \left\langle {{{{\mathbf{t}}}}_j \circ {{{\mathbf{y}}}}} \right\rangle$$

Based on the defined speckle field ${{{\mathbf{y}}}} = {{{\mathbf{Tx}}}} = \mathop {\sum}\nolimits_{i = 1}^N {x_i{{{\mathbf{Te}}}}_i} = \mathop {\sum}\nolimits_{i = 1}^N {x_i{{{\mathbf{t}}}}_i}$ and near-orthogonal properties of speckle fields $\left\langle {{{{\mathbf{t}}}}_i^ \ast \circ {{{\mathbf{t}}}}_j} \right\rangle \approx \left\langle {{{{\mathbf{t}}}}_i^ \ast \circ {{{\mathbf{t}}}}_i} \right\rangle \delta _{ij}$, we can rewrite the second term of Eq. (4) as $x_ix_j^ \ast \left\langle {{{{\mathbf{t}}}}_i^ \ast \circ {{{\mathbf{t}}}}_i} \right\rangle \left\langle {{{{\mathbf{t}}}}_j^ \ast \circ {{{\mathbf{t}}}}_j} \right\rangle$. Then, by substituting Eq. (4) into Eq. (3), we have

5 $$Z_{ij} = x_ix_j^ \ast + \frac{{\left\langle {{{{\mathbf{t}}}}_i^ \ast \circ {{{\mathbf{y}}}}^ \ast } \right\rangle \left\langle {{{{\mathbf{t}}}}_j \circ {{{\mathbf{y}}}}} \right\rangle }}{{\left\langle {{{{\mathbf{t}}}}_i^ \ast \circ {{{\mathbf{t}}}}_i} \right\rangle \left\langle {{{{\mathbf{t}}}}_j^ \ast \circ {{{\mathbf{t}}}}_j} \right\rangle }}$$

The first term of Eq. (5) is the projection matrix of the sample field ${{{\mathbf{xx}}}}^{\dagger}$, which contains the intact sample field information, whereas its second term represents another random matrix. Because the second term approaches zero as the oversampling ratio (*γ* = *M*/*N*) increases, we can directly retrieve the sample field **x** by taking the eigenvector of SSM for large *γ* cases^36^. However, a large *γ* is unlikely because of the finite sampling number (*M*). We want to retrieve as much sample information as possible from the same number of measurements (*M*); therefore, using a *γ* with as small value as possible is generally preferred (e.g., *γ* = 14 in ref. ^39^).

For a small *γ*, an additional error-reduction algorithm should be introduced to compensate for the second-term contribution. Iterative algorithms are usually employed by taking the eigenvector corresponding to the largest eigenvalue of the SSM as an initial guess ${{{\mathbf{x}}}}_i^\prime$. We used the AF^41^. Note that the SSM initialization is similar (but not identical) to the spectral initialization separately proposed in Ref. ^63^.

An additional technique was applied to calculate the SSM. Owing to the intrinsic noise level of the SSM (the second term in Eq. (5)), we observe that a high-frequency signal is barely retrieved in the initial guess ${{{\mathbf{x}}}}_{\rm{i}}^{\prime}$ (Supplementary Fig. S13). Based on this observation, we run the SSM for low-frequency regimes of the samples only, significantly reducing the calculation time.

### Amplitude flow

The initial guess ${{{\mathbf{x}}}}_0^\prime$ for the AF is calculated as ${{{\mathbf{x}}}}^\prime _{{{\mathrm{0}}}} = {{{\hat{\mathbf x}}}}^\prime _i\sqrt {M\left\langle {{{\mathbf{I}}}} \right\rangle /\tau }$, where ${{{\hat{\mathbf x}}}}^\prime _i = {{{\mathbf{x}}}}^\prime _i/\left\| {{{{\mathbf{x}}}}^\prime _i} \right\|$ is the normalized eigenvector corresponding to the largest eigenvalue of the SSM, $\left\langle {{{\mathbf{I}}}} \right\rangle$ is the mean of the measured speckle pattern, and $\tau = {\textstyle{1 \over N}}{{{\mathrm{tr}}}}\left( {{{{\mathbf{T}}}}^{\dagger} {{{\mathbf{T}}}}} \right)$ is a scaling coefficient^63^. Note the physical meaning of $\tau$ is mean transmittance of our system. Since the transmittance of our system is largely defined by the diffuser itself, and barely depends on the sample fields, ${{{\mathbf{t}}}}_i^{\dagger} {{{\mathbf{t}}}}_i \approx {{{\mathbf{t}}}}_j^{\dagger} {{{\mathbf{t}}}}_j$ generally holds. Therefore, we estimated $\tau$ simply by taking the magnitude of the first element of ${{{\mathbf{T}}}}^{\dagger} {{{\mathbf{Te}}}}_1$.

The *k*th AF iteration (for *k* = 0, 1, 2 …) calculates

6 $${{{\mathbf{x}}}}_{k + 1}^\prime = {{{\mathbf{x}}}}_k^\prime - \frac{\mu }{\tau }{{{\mathbf{T}}}}^{\dagger} \left( {{{{\mathbf{y}}}}_k^\prime - {{{\mathbf{w}}}}_k} \right)$$

where *μ* is the step size, ${{{\mathbf{x}}}}_k^\prime$ is the retrieved sample field for the *k*th iteration, ${{{\mathbf{y}}}}_k^\prime = {{{\mathbf{Tx}}}}_k^\prime$ is the corresponding speckle field, and ${{{\mathbf{w}}}}_k$ is the updated speckle field from ${{{\mathbf{y}}}}_k^\prime$ whose amplitude is replaced with the measured speckle amplitude. The *j*th vector component of ${{{\mathbf{w}}}}_k$ is $w_{k,j} = \sqrt {I_j} y_{k,j}/\left| {y_{k,j}} \right|$. The step size *μ* controls the speed of convergence. We used *μ* = 1 throughout the study. We stopped the iteration when the normalized correlation with the previous solution converged at unity, ${\hat{\mathbf x }{}^{\prime}_{k}}^{\dagger} {{{\hat{\mathbf x}}}}_{k - 1}^{\prime}$ >0.9999977, where ${{{\hat{\mathbf x}}}}_k^\prime = {{{\mathbf{x}}}}_k^\prime /\left\| {{{{\mathbf{x}}}}_k^\prime } \right\|$ is the normalized ${{{\mathbf{x}}}}_k^\prime$. This convergence criterion was empirically defined from numerical simulations (see the MATLAB code in Supplementary Text).

### Fourier-transform based TM calculation

As noted, we have *N* = 43,003 and *M* = 778,414 for the results in Figs. 3 and 6. The corresponding size of TM is *M* × *N* = 778,414 × 43,003, which requires ~270 GB of memory with complex single precision. Creating and handling such a large matrix is a tedious and time-consuming process. Because the size of TM is proportional to *N*^2^ for a fixed *γ*, finite computer memory can be a major limiting factor for the FOV or resolution of the retrieved sample field. Therefore, we calculate TM as a series of operations based on a paraxial approximation and Fourier transforms.

Assuming a point source at the origin, we need to determine the propagated field at the axial distance *z* = *L*. The propagated field can be calculated as

7 $$G\left( {x,y,L} \right) = {\int} {e^{i2\pi \left( {ux + vy + wL} \right)}dudv}$$

where (*x*, *y*) are lateral coordinates, and (*u*, *v*, *w*) are the spatial frequencies of (*x*, *y*, *z*), respectively, which satisfy $u^2 + v^2 + w^2 = \lambda ^{ - 2}$. The paraxial approximation $w \approx \lambda ^{ - 1} - {\textstyle{1 \over 2}}\lambda \left( {u^2 + v^2} \right)$ is applied, and the integral is calculated. Equation (7) then becomes

8 $$G\left( {x,y,L} \right) = \frac{1}{{iL\lambda }}e^{i\frac{{2\pi }}{\lambda }L}Q\left( {x,L} \right)Q\left( {y,L} \right)$$

where $Q\left( {x,L} \right) = \exp \left( {i\pi x^2/\left( {\lambda L} \right)} \right)$ is a quadratic phase function. Identical results can be derived from the Rayleigh–Sommerfeld diffraction formula^64^. We omit the *y*-axis hereafter.

Because we have a wavelet from the point source, we can construct the propagation operator of length *L* (P_*L*_) as the convolution form, ${\mathop{\rm P}\nolimits} _L\left[ E \right]\left( x \right) = {\int} {E\left( {x^{\prime}} \right)G\left( {x - x^{\prime},L} \right)dx^{\prime}}$, where $E\left( x \right)$ is an arbitrary field on the plane before the propagation (*z* = 0). Applying Eq. (8) to the convolution, we obtain

9 $${\mathop{\rm P}\nolimits} _L\left[ E \right]\left( x \right) = \frac{1}{{iL\lambda }}e^{i\frac{{2\pi }}{\lambda }L}Q\left( {x,L} \right){\int} {E\left( {x^\prime } \right)Q\left( {x^\prime ,L} \right)e^{ - i\frac{{2\pi xx{\prime}}}{{\lambda L}}}dx^\prime }$$

Note that the integral in Eq. (9) is the Fourier transform of $E\left( x \right)Q\left( {x,L} \right)$, which can be calculated rapidly using the fast Fourier transform (FFT) algorithm. A transmission operation (T) can then be achieved through two propagation operations and the transmission function of the diffuser $t_d\left( x \right)$ – ${\mathop{\rm T}\nolimits} \left[ E \right]\left( x \right) = {\mathop{\rm P}\nolimits} _{L_2}\left[ {t_d \cdot {\mathop{\rm P}\nolimits} _{L_1}\left[ E \right]} \right]\left( x \right)$. As noted, we used a GPU (GeForce GTX 1080 Ti, NVIDIA Corp.) to further accelerate the calculation speed.

### Derivation of resolution limit

Recall that the spatial resolution of our system is defined by $1.22\;L_1\lambda /D_d$. Thus, the resolution limit of a given diffuser diameter (*D*_*d*_) is determined by the minimum *L*_1_ that we can use without violating the working condition of CSI. There are two fundamental working conditions of CSI: (i) the measured image must be a speckle pattern, and (ii) the oversampling ratio (*γ*) should be larger than the minimum oversampling ratio for stable field retrieval (*γ*_min_).

The condition (i) is related to Eq. (4), and it is the exact reason why we require a diffuser for CSI. However, despite the use of a diffuser, this condition may fail if *L*_1_ is so small that the sample field can produce a finer focus on a diffuser surface than the diffuser feature size. More precisely, the spatial bandwidth of the sample field on a diffuser surface should be smaller than the modulating bandwidth of the diffuser (BW_*d*_), $D_s/\left( {L_1\lambda } \right) \,<\, {{{\mathrm{BW}}}}_d$. Because the BW_*d*_ can be characterized by the maximum divergence angle of the diffuser (*θ*_*d*_), ${{{\mathrm{BW}}}}_{\it{d}} = 2\theta _{\it{d}}/\lambda$, we get the first condition of *L*_1_,

10 $$L_1 > \frac{{D_s}}{{2\theta _d}}$$

In this work, we define *θ*_*d*_ based on the far-field diffraction pattern of the diffuser (Fig. 2d). Considering the central circular plateau as a conservative effective angle deflection distribution of diffuser, we get $\theta _d = 1.22\;\lambda /D_h$ from the first zero of sombrero function.

The condition (ii), $\gamma \,>\, \gamma _{\min }$, is related to the reconstruction stability of CSI as explored in the previous works^36,39^. The *γ* is defined by $M/N$, where *N* and *M* are the numbers of spatial modes of the sample and speckle fields, respectively. The *γ*_min_ is an experimental parameter that is largely depending on the noise level of an imaging system. Even in noiseless situations, it is empirically known that *γ*_min_ ≥ 4 is required for stable reconstructions^36,39,41^. In this work, we conservatively set *γ*_min_ = 7 considering potential practical noises and imperfections. The *M* and *N* can be quantified by calculating SBP^60^. For example, the spatial and reciprocal diameters of the sample field are *D*_*s*_ and $D_d/\left( {L_1\lambda } \right)$; therefore, the *N* becomes

11 $$N = \frac{\pi }{4}D_s^2 \cdot \frac{\pi }{4}\left( {\frac{{D_d}}{{L_1\lambda }}} \right)^2$$

Similarly, the *M* becomes

12 $$M = \frac{\pi }{4}D_{{{{\mathrm{spk}}}}}^2 \cdot \frac{\pi }{4}\left( {\frac{{D_d}}{{L_2\lambda }}} \right)^2$$

where *D*_spk_ is the diameter of the speckle pattern on the detector plane.

Because we considers the lower bound of *L*_1_, the synthetic aperture regime should be considered here to define the oversampling ratio correctly (Fig. 5). When a sample diffraction angle exceeds *θ*_*d*_, the diffuser fails to collect all the sample diffraction into a single point (Fig. 5, inset). In other words, a single point on the detector plane cannot see the entire diameter of the diffuser, but only its portion. If two points on the detector plane see the completely separated portions, they are no longer relevant to each other. Therefore, the diffuser should be regarded as the composition of the sub-diffusers rather than the whole. To calculate Eqs. (11) and (12) for sub-diffusers, sub-diffuser diameter ($D_d^{{{{\mathrm{sub}}}}}$) and effective diameter of a speckle pattern for a sub-diffuser ($D_{s{{{\mathrm{pk}}}}}^{{{{\mathrm{sub}}}}}$) are need to be defined first.

The $D_d^{{{{\mathrm{sub}}}}}$ can be calculated from the maximum diameter of the diffuser that can be seen from the single point of the detector (Fig. 5, inset). A momentum (or angle) conservation equation can be constructed,

13 $$\frac{{\left( {D_d^{{{{\mathrm{sub}}}}} - D_s} \right)}}{{2L_1}} + \frac{{D_d^{{{{\mathrm{sub}}}}}}}{{2L_2}} = \theta _d$$

The first and second terms in left-hand side of Eq. (13) are the diverging angle starting from the edge of sample FOV before the diffuser, and the converging angle to a single point of the detector after the diffuser, respectively. The addition of the two angles become *θ*_*d*_ for the largest possible sub-diffuser diameter $D_d^{{{{\mathrm{sub}}}}}$. Solving Eq. (13) for $D_d^{{{{\mathrm{sub}}}}}$, we can define the sub-diffuser diameter

14 $$D_d^{{{{\mathrm{sub}}}}} = \frac{{L_2}}{{L_1 + L_2}}\left( {D_s + 2L_1\theta _d} \right)$$

for the given parameters, *D*_*s*_, *L*_1_, and *L*_2_. When $D_d^{{{{\mathrm{sub}}}}} \,<\, D_d$, it is the synthetic aperture regime, and the sub-diffusers should be considered; otherwise ($D_d^{{{{\mathrm{sub}}}}}\, >\, D_d$), it is the single-aperture regime, Eqs. (13) and (14) become invalid, and the diffuser is regarded as a whole (Fig. 5). Note that the dashed line in Fig. 5 is $D_d^{{{{\mathrm{sub}}}}} = D_d$, which is the boundary between the two regimes.

The $D_{s{{{\mathrm{pk}}}}}^{{{{\mathrm{sub}}}}}$ can be estimated from the projection of the sub-diffusers onto the detector plane (Supplementary Fig. S14),

15 $$D_{s{{{\mathrm{pk}}}}}^{{{{\mathrm{sub}}}}} = \left( {1 + \frac{{L_2}}{{L_1}}} \right)D_d^{{{{\mathrm{sub}}}}}$$

For a single sub-diffuser, Eq. (15) may seem irrational because it completely ignores the diffraction portion from the diffuser. However, we find such diffractive FOV overlapping between adjacent sub-diffusers is a mutual effect, and becomes insignificant when determining the effective FOV.

Substituting Eqs. (14) and (15) to Eqs. (11) and (12), we get the oversampling ratio for sub-diffusers, $\gamma = \left( {1 + 2L_1\theta _d/D_s} \right)^2$, and the condition (ii) finally becomes

16 $$L_1 > \left( {\sqrt {\gamma _{\min }} - 1} \right)\frac{{D_s}}{{2\theta _d}}$$

Note that Eq. (16) includes Eq. (10) if $\gamma _{\min } \ge 4$. Because $\gamma _{\min } \ge 4$ is empirically known as the oversampling criteria of noiseless situations^36,39,41^, we can conclude that Eq. (16) solely determines the lower bound of *L*_1_. The resolution limit [Eq. (1)] is acquired by substituting Eq. (16) to the definition of spatial resolution ($\delta x = 1.22\;L_1\lambda /D_d$) with $\theta _d = 1.22\;\lambda /D_h$.

### Criteria for available *L*_1_ and *L*_2_

In Fig. 5, there are three criteria for available *L*_1_ and *L*_2_ related to the oversampling condition (the purple line), sampling resolution (the blue line), and detector size (the red line). The purple line, derived from the oversampling condition, is elaborated in the Derivation of resolution limit section in Methods. As shown in Eq. (16), it provides the lower bound of *L*_1_. Unlike the other two criteria, this is a fundamental criterion that is irrelevant to practical sampling conditions.

The blue line in Fig. 5 is introduced to maintain a speckle grain size bigger than the practical sampling resolution (*p*). More precisely, the spatial bandwidth of the speckle field on the detector plane should be smaller the bandwidth of detector, $D_d/\left( {L_2\lambda } \right) < 1/p$, which provides the lower bound of *L*_2_,

17 $$L_2 > \frac{p}{\lambda }D_d$$

In the single-aperture regime, this criterion is independent to *L*_1_ as indicated by the horizontal line in Fig. 5. In the synthetic aperture regime, the diffuser diameter (*D*_*d*_) in Eq. (17) should be replaced to the sub-diffuser diameter ($D_d^{{{{\mathrm{sub}}}}}$), which is a function of *L*_1_ and *L*_2_ as defined in Eq. (14). Thus, this criterion becomes a function of both *L*_1_ and *L*_2_ in the synthetic aperture regime (Fig. 5). The *p* can be defined either by the detector pixel size or by the resolution limit of the detection system. In this work, we used the two camera pixel sizes on the scintillator (*p* = 1.3 μm, considering the ×10 microscope magnification). We set the *p* to two pixels (rather than one) to acquire the full bandwidth of the intensity speckle pattern, which is twice the bandwidth of the speckle field in reciprocal space^61^. This is particularly important in the image pre-processing steps.

The red line in Fig. 5 is introduced to fulfill the oversampling condition in a given detector having a finite sensor size. Even if the fundamental criterion (the purple line in Fig. 5) is satisfied, the oversampling condition ($\gamma \,>\, \gamma _{\min }$) can still be violated if the practical detector size (*F*) is too small. Thus, we need to introduce one additional practical condition,

18 $$F \,>\, D_{{{{\mathrm{spk}}}}}$$

where *D*_spk_ is the diameter of a speckle pattern on the detector plane. This is an obvious condition because we cannot acquire the speckle patterns generated outside of detector. In the single-aperture regime, the oversampling condition can simply be defined by Eqs. (11) and (12),

19 $$\gamma = \left( {\frac{{D_{{{{\mathrm{spk}}}}}}}{{D_s}}\frac{{L_1}}{{L_2}}} \right)^2 \,>\, \gamma _{\min }$$

Substituting Eq. (18) to Eq. (19), we get

20 $$\frac{F}{{D_s}}\frac{{L_1}}{{\sqrt {\gamma _{\min }} }} > L_2$$

which provides an upper bound of *L*_2_ as presented in Fig. 5. In the synthetic aperture regime, although the oversampling condition of internal sub-diffusers is already considered in Eq. (16), the oversampling condition of the outermost sub-diffuser need to be considered separately as there are no adjacent sub-diffusers on one side (Supplementary Fig. S14). As shown in Supplementary Fig. S14, the required detector diameter for internal sub-diffusers should be $D_{s{{{\mathrm{pk}}}}}^{{{{\mathrm{sub}}}}}$ multiplied by the number of sub-diffusers on the centerline of the diffuser, $\left( {D_d - D_d^{{{{\mathrm{sub}}}}}} \right)/D_d^{{{{\mathrm{sub}}}}}$, which becomes $\left( {1 + L_2/L_1} \right)\left( {D_d - D_d^{{{{\mathrm{sub}}}}}} \right)$ according to Eq. (15). If we define $D_d^{{{{\mathrm{out}}}}}$ as the required speckle pattern diameter of the outermost sub-diffuser, Eq. (18) in the synthetic aperture regime becomes

21 $$F \,>\, D_d^{{{{\mathrm{out}}}}} + \left( {1 + \frac{{L_2}}{{L_1}}} \right)\left( {D_d - D_d^{{{{\mathrm{sub}}}}}} \right)$$

Since Eq. (21) should converge to Eq. (20) as $D_d^{{{{\mathrm{sub}}}}} \to D_d$, we can deduce that $D_d^{{{{\mathrm{out}}}}} = \sqrt {\gamma _{\min }} D_sL_2/L_1$. Because $D_d^{{{{\mathrm{sub}}}}}$ is also a function of *L*_1_ and *L*_2_ [Eq. (14)], Eq. (21) is a nonlinear curve on the *L*_1_-*L*_2_ plane as shown in Fig. 5. In this work, *F* is determined by the sensor size of the camera (2048 × 2048), which is 1331 μm on one side, considering the ×10 microscope magnification.

## Supplementary information

Supplemental Information

## Data Availability

The data are available from the corresponding authors upon reasonable request.
