# Supplementary material for: Direct high-resolution X-ray imaging exploiting pseudorandomness
Source: Light Sci Appl. 2023 Apr 6;12:88. doi: 10.1038/s41377-023-01124-3 (PMC10079858; doi:10.1038/s41377-023-01124-3)
Supplement: Supplementary file 1 — Supplemental Information [file 41377_2023_1124_MOESM1_ESM.docx]

Supplementary Information for:

Direct high-resolution X-ray imaging exploiting pseudorandomness

KyeoReh Lee, Jun Lim, Su Yong Lee, and YongKeun Park

**Figure S1 | Diffuser hole size variation and consequent changes in speckle patterns.** The speckle patterns are numerically generated from the diffusers having same hole positions but different hole size variation. The hole size was set as a random variable with a normal distribution, whose mean is fixed at 300 nm. The hole size variation is controlled by adjusting the standard deviation (σ) of the probability distribution. From left, each column represents σ = 0, 10, 20, 30, and 40 nm cases. The top row shows the changes in diffraction lobe shape. The bottom row is the magnified version of the black rectangular areas in the top row images, and shows insignificant variations in granular patterns.

**Figure S2 | Diffuser roll angle deviation and consequent changes in field retrieval results.** The diffuser roll angle is numerically deviated from the calibrated angle. The measured speckle pattern for a 50-nm half-pitch grating (Fig. 3a) is used with an identical reconstruction flow except for the diffuser roll angle. From left, each column represents 0°, 0.1°, 0.5°, 1.0°, and 2.0° angle deviation cases. The top row presents corresponding retrieved phase images. The bottom row shows the phase difference images from the calibrated one, which visualizes image quality degradation.

**Figure S3 | Field retrieval flow chart of coherent speckle-correlation imaging.** The sample field is retrieved utilising the measured intensity speckle pattern, calculated transmission matrix, and the randomness of speckle. The speckle-correlation scattering matrix (SSM) is constructed to provide the initial guess. The sample field is retrieved through the amplitude flow (AF). Please see the Methods for details.

**Figure S4 | low-resolution reconstructions. a-c**, The 100-nm half-pitch grating results. **d-f**, The 200-nm half-pitch grating results. The amplitude (**a** and **d**) and phase (**b** and **e**) images, and the reciprocal space (**c** and **f**) are shown. The sample fields are retrieved using the sample speckle pattern (with Fig. 2), but with a reduced reciprocal space sampling domain. The central 25-μm diameter portion of the diffuser is considered in the reconstruction algorithm. Reconstruction time is reduced to 9.5 s.

**Figure S5 | Larger sample field-of-view reconstructions.** **a-c**, The 100-nm half-pitch grating results. **d-f**, The 200-nm half-pitch grating results. The amplitude (**a** and **d**) and phase (**b** and **e**) images, and the reciprocal space (**c** and **f**) are shown. The real space domain is set to be 10 μm diameter, while the size of the experimental field stop remains still (~3 μm diameter). Due to the criteria (see Fig. 5), *L*_1_ =13 mm and *L*_2_ = 180 mm are used for these results. The corresponding system resolution is ~36 nm.

**Figure S6 | Field retrieval fidelity comparison with coherent modulation imaging (CMI).** An experimentally measured a 400-nm half-pitch grating sample with a 3-μm diameter field stop is reconstructed in a 10-μm diameter sample field-of-view. **a**, Proposed reconstruction algorithm is used. This is an identical result with Fig. S5 but has been brought in for easy comparison. **b-d**, Three reconstruction trials with CMI algorithm proposed in Ref. [23] of manuscript. Due to the random initial points, we got significantly different results despite the use of identical data and parameters. The step size α = 0.5 is used. Iteration sequence stops when the convergence criteria are satisfied or the 1,000th iteration is finished.

**Figure S7 | The intermediate results of Fig. 3b.** **a**, The measured speckle pattern. **b**, The speckle pattern after the image preprocessing (the registration of position and rotation and deconvolution). **c**, The retrieved speckle pattern. **d,** The initial guess from the speckle-correlation scattering matrix (SSM). **e**, The retrieved sample field after the amplitude flow (AF) iterations. **f**, the refocused sample field via numerical propagation from the retrieved sample field (**e**). For **d-e**, the normalized amplitude images, phase images, and reciprocal spaces are shown above, middle, below, respectively.

**Figure S8 | The intermediate results of Fig. 3e.** **a**, The measured speckle pattern. **b**, The speckle pattern after the image preprocessing (the registration of position and rotation and deconvolution). **c**, The retrieved speckle pattern. **d,** The initial guess from the speckle-correlation scattering matrix (SSM). **e**, The retrieved sample field after the amplitude flow (AF) iterations. **f**, the refocused sample field via numerical propagation from the retrieved sample field (**e**). For **d-e**, the normalized amplitude images, phase images, and reciprocal spaces are shown above, middle, below, respectively.

**Figure S9 | The intermediate results of Fig. 3f.** **a**, The measured speckle pattern. **b**, The speckle pattern after the image preprocessing (the registration of position and rotation and deconvolution). **c**, The retrieved speckle pattern. **d,** The initial guess from the speckle-correlation scattering matrix (SSM). **e**, The retrieved sample field after the amplitude flow (AF) iterations. **f**, the refocused sample field via numerical propagation from the retrieved sample field (**e**). For **d-e**, the normalized amplitude images, phase images, and reciprocal spaces are shown above, middle, below, respectively.

**Figure S10 | The effect of consistent noise signal**. **a-h**, Field retrieval results with (**a**-**d**) and without (**e**-**h**) noise signal at the left-bottom corner of the speckle pattern: the speckle patterns (**a** and **e**), the reciprocal spaces of retrieved field (**b** and **f**), the retrieved amplitude image (**c** and **g**), and the retrieved phase images (**d** and **h**). Figures **a**-**d** are identical to those in Fig. S9 but have been brought in for easy comparison.

**Figure S11 | Individual field retrieval results in Fig. 6(a).** **a**, amplitude images. **b**, phase images. Stitched images are presented (left), and six scanning points are presented among 91 (right). Notice spacing between scanning points are uneven due to the practical inaccuracy of the used stage.

**Figure S12 | Point spread function (PSF) and optical transfer function (OTF). a**, The PSF we measured. **b**, the magnitude of corresponding OTF (or the reciprocal space of **a**). **c**, azimuthally averaged **b** comparing with the ideal case.

**Figure S13 | Low-resolution trick for speckle-correlation scattering matrix (SSM) calculation.** Two initial guesses are shown in **b-f**. **b** and **d** are the amplitude and phase images of the initial guess using the entire speckle pattern (**a**). **c** and **f** are the amplitude and phase images of the initial guess using the cropped speckle pattern (the subset (i) of **a**).

**Figure S14 |** Two aperture regimes. **a**-**b**, Single- (**a**) and synthetic (**b**) aperture regimes are drawn. Please find the detailed explanation of figures in the Methods. For better visualization, the deflection angles are hugely exaggerated, diffuser and detector are drawn curved, and field and aperture stops are ignored here. For the synthetic aperture regime, five non-overlapping sub-diffusers are drawn as examples.

**Supplementary Methods**

**MATLAB^®^ code for coherent speckle-correlation imaging (CSI)**

% Written by KyeoReh Lee, 2021-08-10

% lee.kyeo@gmail.com

%% initialization

gamma = 5; % starts to fail when gamma < 4

N1d = 32;

% AF iteration parameters

iterMax = 1000;

corrCrit = 10^-6;

stepSize = 1;

%% sample field definition

% other x0_2d can be used.

% random input: x0_2d = randn(N1d) + 1i*randn(N1d);

% point source: x0_2d = zeros(N1d); x0_2d(randperm(N1d^2,1)) = 1;

% normal planewave : x0_2d = ones(N1d);

abs0	= double( imread('cameraman.tif') );

angle0 = sum( double( imread('peppers.png') ) , 3);

abs0	= imresize(abs0, [N1d, N1d]);

angle0 = 5 * imresize(angle0, [N1d, N1d]) / max(angle0(:));

x0_2d = abs0 .* exp(1i*2*pi*angle0);

%% speckle field generation

x_0 = x0_2d(:);

N	= N1d^2;

M	= gamma * N;

TM = randn(M, N) + 1i * randn(M, N);

y_0 = TM * x_0;

%% measurement

Iyy = abs(y_0).^2; % we cannot measure the phase

%% SSM

% Rerences:

% Lee, KyeoReh, el al. Nature communications 7.1 (2016): 1-7.

% Lee, KyeoReh, el al. Physical Review Applied 12.2 (2019): 024003.

% Baek, YoonSeok, et al. Sensors 20.11 (2020): 3147.

sigma	= mean(abs(TM).^2, 1);

numerator	= TM' * (( Iyy-mean(Iyy) ) .* TM);

denominator = sigma' * sigma;

Zmatrix	= numerator ./ denominator; % Z matrix calculation.

[Xretv0, ~] = eigs(double(Zmatrix), 1); % Select the eigenvector of the largest eigenvalue.

Xretv0_fidelity = abs( x_0'*Xretv0 / norm(x_0) / norm(Xretv0) );

fprintf('SSM init. guess fidelity : \t%.2f\n', Xretv0_fidelity);

%% Amplitude flow iteration

% Rerences:

% Candes, Emmanuel J., et al. IEEE Transactions on Information Theory 61.4 (2015): 1985-2007.

% Wang, Gang, et al. IEEE Transactions on Information Theory 64.2 (2017): 773-794.

% initial guess for the AF

tau	= trace(TM'*TM)/N;

Xiter = Xretv0 / norm(Xretv0) * sqrt( M * mean(Iyy) / tau );

% measured amplitude

psi = sqrt(Iyy);

% iteration run

Xcorr_iter = zeros(iterMax, 1);

Xfinal_fidelity = zeros(iterMax, 1);

figure(1),

for kk = 1:iterMax

Yiter = TM * Xiter;

Witer = psi .* Yiter ./ abs(Yiter);

gradTerm = -stepSize/tau * (TM' * ( Yiter - Witer ));

Xbefore = Xiter;

Xiter	= Xiter + gradTerm;

Xcorr_iter(kk) = log10( abs( Xbefore' * Xiter /norm(Xbefore) /norm(Xiter) ) );

Xfinal_fidelity(kk)	= abs( x_0' * Xiter /norm(x_0) /norm(Xiter) );

% show

yyaxis left, plot(Xcorr_iter(1:kk)),axis square;

ylabel('log_{10}(abs( Correlation with the last iteration ))')

yyaxis right, plot(Xfinal_fidelity(1:kk));

ylabel(' Correlation with the Ground truth ')

xlabel('iteration #')

drawnow;

% stop?

iterStopTF = -Xcorr_iter(kk) < corrCrit;

if iterStopTF

break;

end

end

Xfinal	= Xiter;

Xcorr_iter = Xcorr_iter(1:kk);

Xfinal_fidelity	= Xfinal_fidelity(1:kk);

fprintf('Amplitude flow (iter# = %d) fidelity : %f\n', kk, Xfinal_fidelity(kk));

%% Results

Xretv0_2d = reshape(Xretv0, [N1d, N1d]);

Xfinal_2d = reshape(Xfinal, [N1d, N1d]);

% amplitude nomalization and global phase matching

Xretv0_2d = Xretv0_2d * exp(-1i*angle(x_0'*Xretv0)) * norm(x_0)/norm(Xretv0);

Xfinal_2d = Xfinal_2d * exp(-1i*angle(x_0'*Xfinal));

% show

figure(2)

subplot(231), imagesc(abs(x0_2d)), axis image; title('Ground truth')

h = subplot(231); absCLim = h.CLim;

subplot(232), imagesc(abs(Xretv0_2d), absCLim), axis image; title('initial guess from the SSM')

subplot(233), imagesc(abs(Xfinal_2d), absCLim), axis image; title('Retrieved sample field')

subplot(234), imagesc(angle(x0_2d),[-pi,pi]), axis image; title('upper: abs, lower: angle')

subplot(235), imagesc(angle(Xretv0_2d),[-pi,pi]), axis image; title(['Fidelity (init. guess): ', num2str(Xretv0_fidelity,'%.2f')])

subplot(236), imagesc(angle(Xfinal_2d),[-pi,pi]), axis image; title(['Fidelity (final): ', num2str(Xfinal_fidelity(kk),'%.2f')])
